# Defining critical illness using immunological endotypes in patients with and without of sepsis: A cohort study

**DOI:** 10.21203/rs.3.rs-2874506/v1

**Published:** 2023-05-08

**Authors:** Jeremy A. Balch, Uan-I Chen, Oliver Liesenfeld, Petr Starostik, Tyler J. Loftus, Philip A. Efron, Scott C. Brakenridge, Timothy E. Sweeney, Lyle L. Moldawer

**Affiliations:** University of Florida College of Medicine; Inflammatix, Inc; Inflammatix, Inc; UF Health Medical Laboratory at Rocky Point, University of Florida College of Medicine; University of Florida College of Medicine; University of Florida College of Medicine; University of Florida College of Medicine; Inflammatix, Inc; University of Florida College of Medicine

**Keywords:** transcriptomics, biomarkers, ICU, outcomes, sepsis

## Abstract

**Background:**

Sepsis is a heterogenous syndrome with limited therapeutic options. Identifying characteristic gene expression patterns, or endotypes, in septic patients may lead to targeted interventions. We investigated whether patients admitted to a surgical ICU with sepsis and with high risk of mortality express similar endotypes to non-septic, but still critically ill patients using two multiplex transcriptomic metrics obtained both on admission to a surgical intensive care unit (ICU) and at set intervals.

**Methods:**

We analyzed transcriptomic data from 522 patients in two single-site, prospective, observational cohorts admitted to surgical ICUs over a 5-year period ending in July 2020. Using an FDA-cleared analytical platform (nCounter FLEX^®^, NanoString, Inc.), we assessed a previously validated 29-messenger RNA transcriptomic classifier for likelihood of 30-day mortality (IMX-SEV-3) and a 33-messenger RNA transcriptomic endotype classifier. Clinical outcomes included all-cause (in-hospital, 30-, 90-day) mortality, development of chronic critical illness (CCI), and secondary infections. Univariate and multivariate analyses were performed to assess for true effect and confounding.

**Results:**

Sepsis was associated with a significantly higher predicted and actual hospital mortality. At enrollment, the predominant endotype for both septic and non-septic patients was *adaptive*, though with significantly different distributions. *Inflammopathic* and *coagulopathic* septic patients, as well as *inflammopathic* non-septic patients, showed significantly higher frequencies of secondary infections compared to those with adaptive endotypes (p<0.01). Endotypes changed during ICU hospitalization in 57.5% of patients. Patients who remained *adaptive* had overall better prognosis, while those who remained *inflammopathic* or *coagulopathic* had worse overall outcomes. For severity metrics, patients admitted with sepsis and a high predicted likelihood of mortality showed an *inflammopathic* (49.6%) endotype and had higher rates of cumulative adverse outcomes (67.4%). Patients at low mortality risk, whether septic or non-septic, almost uniformly presented with an adaptive endotype (100% and 93.4%, respectively).

**Conclusion:**

Critically ill surgical patients express different and evolving immunological endotypes depending upon both their sepsis status and severity of their clinical course. Future studies will elucidate whether endotyping critically ill, septic patients can identify individuals for targeted therapeutic interventions to improve patient management and outcomes.

## Introduction

Sepsis remains one of the most common causes of mortality and morbidity in critically ill patients, affecting as many as 50 million individuals annually with case mortality rates as high as 40% [1]. Earlier recognition and near-universal implementation of sepsis protocols have improved in-hospital clinical outcomes; however, targeted therapies remain elusive [2–4].

Sepsis is defined as a dysregulated host immune response to infection resulting in life-threatening organ dysfunction [5, 6]. However, inherent to this definition is a wide range of insults and trajectories of physiologic decline. This disease heterogeneity likely explains the lack of efficacy in previous randomized controlled trials employing immune modulating therapeutics [7–11]. To address this heterogeneity, efforts have been made to classify patients based on constellations of observable characteristics and commonly available laboratory values, also called phenotypes [12–15]. However, phenotypes based on these clinical variables may not accurately discriminate differences in the underlying disease mechanisms, also called endotypes. Thus, efforts at phenotyping have not led to substantial changes in patient care or outcomes [16, 17].

Multiplex metabolomics, proteomics, and transcriptomics offer the potential to reveal a spectrum of sepsis endotypes, both illuminating common underlying mechanisms for immunological dyscrasia and providing potential therapeutic targets. Previous research has identified 2–5 subgroups in diagnosed sepsis, though they vary with regards to endotype domains, data sources, classification algorithms, statistical methodology, duration of observations, and stated goals [18–24].

In this study, we apply 29- and 33-gene transcriptomic signatures to simultaneously classify sepsis severity and endotype, respectively [23, 25–27]. These transcriptomic signatures were originally validated in non-surgical patients with bacterial or viral sepsis, and were classified into *adaptative, inflammopathic*, and *coagulopathic* endotypes based on gene ontology analysis [23]. We investigate whether patients admitted to a surgical ICU with sepsis and with high risk of mortality would express similar endotypes to non-septic, but still critically ill patients at-risk of developing sepsis. We examine how these endotypes evolve overtime in critically ill patients who either rapidly recover or develop complications during their clinical course.

## Materials and Methods

### Study Designs:

**Figure 1:**
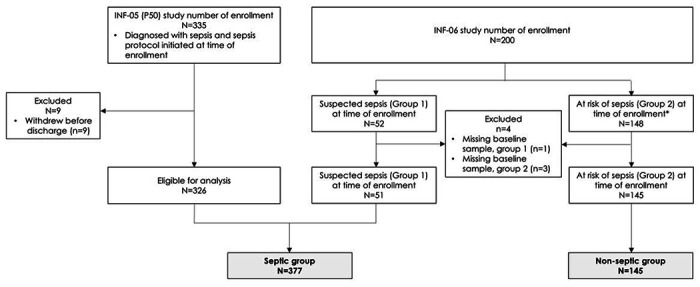
Study Inclusion Criteria. Study population was derived from two single-site, prospective, observational studies that enrolled a total of 522 patients admitted to a non-cardiac, surgical ICUs. *All data points are at time of enrollment. Therefore, the 11 crossover patients were included in the non-septic group since they were not septic at the time of enrollment.

This study performed transcriptomic analyses on samples from two single-site, prospective, observational cohorts that enrolled a total of 522 patients admitted to non-cardiac, surgical ICUs and were classified as either (1) critically ill patients with a diagnosis of sepsis (Septic), or (2) non-septic critically-ill patients, at high risk for subsequently developing sepsis (At-Risk or Non-Septic; Fig. 1) [25, 26]. Data and additional samples were obtained from the University of Florida CTSA Biorepository, a resource available to the scientific community [28]. In the first study (INF-05) [26], the parent cohort included 363 patients admitted to a surgical ICU between January 2015 and January 2020 with a diagnosis of sepsis (NCT02276417). Sepsis cohort inclusion criteria were: 1) age greater than or equal to 18 years, 2) clinical diagnosis of sepsis as defined by 2001 consensus guidelines, and 3) entrance into the electronic health record (EHR)-based sepsis clinical management protocol. Although prospectively enrolled using 2001 sepsis consensus criteria, these patients were retrospectively readjudicated and reclassified using Sepsis-3 consensus definitions [5, 29]. Detailed descriptions of the inclusion and exclusion criteria are contained in the **Supplemental Methods and Supplemental Fig. 1**. Our study was performed in accordance with the STROBE guidelines.

The second prospective diagnostic and prognostic study (INF-06) was conducted between July 2020 and July 2021 [25] and enrolled two cohorts of critically ill patients at the time of surgical ICU admission (NCT04414189). One cohort included patients with a suspected diagnosis of sepsis admitted to the ICU for protocolized sepsis management, as in previously described study. Sepsis was defined according to Sepsis-3 criteria. The second cohort included critically ill patients without sepsis (e.g., severely injured trauma patients, post-operative patients, patients admitted directly to ICU from emergency department, see **Supplementary Table 1**). Detailed inclusion and exclusion criteria, study design, and cohort flow are contained in Fig. 1 and the **Supplemental Methods and Results**.

In both studies, all enrolled subjects underwent post hoc adjudication by physician-investigators within one week of cohort enrollment to confirm sepsis diagnosis, severity, and source. Hospital-acquired secondary infections were adjudicated by physician-investigators during primary data/chart review utilizing current United States Centers for Disease Control definitions and guidelines [10].

Individual clinical outcome variables included all-cause (in hospital, 30-, 90-day) mortality, development or absence of chronic critical illness (CCI), secondary infections, and poor discharge disposition. Inpatient clinical trajectory was defined as “early death,” “rapid recovery”, or “CCI”. CCI was defined as an ICU length of stay greater than or equal to 14 days with evidence of persistent organ dysfunction (SOFA score ≥ 2) [30, 31]. Hospitalized patients who died after an ICU length of stay greater than 14 days from the index hospitalization were also classified as CCI. Rapid recovery patients were those discharged from the ICU within 14 days following resolution of organ dysfunction. Poor disposition was defined as discharge to a skilled nursing facility, long-term acute care facility, or hospice.

### Sample collections.

Blood samples were collected in PAXgene^™^ blood RNA tubes within 12–24 hours of ICU admission and were stored at ^−8^0°C for subsequent analysis. In the second study, additional blood samples were also collected on ICU days 4 and 7 and weekly thereafter during ICU stay (**Supplemental Methods)**. RNA was extracted with the RNeasy® Plus Micro Kit (QIAGEN, Germantown, MD). The IMX-SEV-3 severity and the 33-mRNA endotyping classifiers were quantitated simultaneously from 200 ng of RNA input using the 510(k)-cleared NanoString nCounter FLEX^™^ profiler (NanoString, Seattle, WA) according to a validated standard operating protocol in a Clinical Laboratory Improvement Amendments-certified diagnostic laboratory (UF Health Medical Laboratories at Rocky Point, Gainesville, FL) by licensed laboratory technicians.

### Severity and Endotype classification:

The likelihood of an adverse clinical outcome (in-hospital, 30- and 90-day mortality, development of CCI and discharge disposition) was estimated by a 29 host-messenger RNA (mRNA) test, (IMX-SEV-3, Inflammatix Inc., Burlingame, CA) that uses a machine learning algorithm to report results as both a continuous variable and stratified “risk bands” to meet clinically actionable performance thresholds: “low,” “moderate,” and “high” likelihood of 30-day mortality (see **Supplementary Fig. 2**) [32]. Severity classification was performed using supervised multi-layer perceptron (MLP) models as previously described [27].

Classification into three endotypes was computed from the whole blood expression of 33 host immune mRNAs using a previously published classifier [23, 25, 33]. These endotypes–*adaptive, inflammopathic*, and *coagulopathic*–were derived from the difference of geometric means of gene expression for each of three modules. The inflammopathic module comprises the expression of *ARG1, LCN2, LTF, OLFM4*, and *HLA-DMB*; the coagulopathic module comprises *KCNMB4, CRISP2, HTRA1, PPL, RHBDF2, ZCCHC4, YKT6, DDX6, SENP5, RAPGEF1, DTX2*, and *RELB*; and the adaptive module comprises *YKT6, PDE4B, TWISTNB, BTN2A2, ZBTB33, PSMB9, CAMK4, TMEM19, SLC12A7, TP53BP1, PLEKHO1, SLC25A22, FRS2, GADD45A, CD24, S100A12*, and *STX1A* expression. An overall endotype assignment for each subject was calculated using a 3-class logistic regression model which takes as input the three modules and generates a probability of endotype assignment (for each subject, the total probability [p(Inflammopathic) + p(Adaptive) + p(Coagulopathic)] sums to 1). Each sample is assigned an endotype according to the highest probability. Numerical values are presented in the **Supplemental Table 2** [23].

Total leukocyte and absolute lymphocyte counts (ALCs) were determined at the University of Florida Health Clinical and Diagnostic Laboratories. Plasma IL-6 levels were determined using the Luminex MagPix^®^ platform (Austin, TX).

#### Statistical analysis

Descriptive data are presented as frequencies and percentages or means and standard deviations (SD). The Fisher exact test or Pearson’s Chi-squared test and t-test were used for comparison of categorical and continuous variables, respectively. All significance tests were two sided, with a raw p ≤ 0.05 considered statistically significant. Univariable and multivariable logistic regressions were performed, controlling for age, sex, WBC, IL-6, SOFA, endotype, Charlson Comorbidity Index, and septic status. Analyses were performed using the R Project statistical package, version 4.2.0 (R Project for Statistical Computing).

#### Study Approval

Ethics approval was obtained from the University of Florida Institutional Review Board (IRB#201400611). Informed consent was obtained from each subject or their surrogate decision-maker. Self-reported or proxy-reported race and ethnicity category data were collected as per National Institutes of Health reporting guidelines and requirements.

## Results

### Septic and Non-Septic Cohorts

The overall analytic cohort consisted of 522 critically ill patients from the two consecutive, prospective observational studies (Fig. 1). Prediction of sepsis severity and endotype analyses were conducted on 377 septic and 145 non-septic patients within 24 hours of ICU admission (Table 1). A subset of septic (N = 51) and all non-septic (N = 145) patients had repeat blood sampling at designated intervals over their ICU stay. Three hundred and twenty-six (86%) septic patients were drawn from the initial cohort (INF-05) and all at-risk patients were drawn from the second cohort (INF-06) [25, 26]. Demographics of included patients are shown in Table 1, while Table 2 shows outcomes, endotypes, and severity predictions of the two critically ill cohorts.

As expected, critically ill patients admitted to the ICU with sepsis had significantly higher SOFA and Charlson Comorbidity scores compared to the non-septic cohort, indicating more severe organ dysfunction and greater number of comorbidities. As shown in Table 2, poorer outcomes were observed among the septic cohort, including a higher incidence of secondary infection (30.2 vs 8.3%, p < 0.001), development of CCI (32.4 vs 6.9%, p < 0.001), poor discharge disposition (40.4 vs 16.0%, p < 0.001), in-hospital mortality (7.4 vs 2.1%, p = 0.02), and 30- (10.2 vs 4.1%, p = 0.03) and 90-day (16.8 vs 5.5%, p < 0.001) mortality.

### Endotype distributions and outcomes

Endotype distributions were significantly different between septic and non-septic groups (Table 2). In both septic and non-septic cohorts, the adaptive endotype was most frequent, although it was more common in non-septic patients (40.1% vs 51%). The inflammopathic endotype was second most common in septic patients and third in non-septic patients (34.2 vs 15.9%) (Table 2). However, septic patients (n = 377) had different clinical outcomes depending upon their endotype at admission (Table 3). Inflammopathic and coagulopathic septic patients had a significantly higher frequency of secondary infections (37% each) compared to septic patients with an adaptive endotype (20%, p < 0.01). Similar increases in the frequency of secondary infections were seen in the inflammopathic non-septic patients (26%) versus patients with coagulopathic (4%) or adaptive (5%) endotypes (p < 0.01). Thirty-day mortality, CCI, and adverse discharge disposition followed similar trends, although did not reach statistical significance.

To examine whether endotype at baseline is associated with different patient outcomes, a multivariable logistic regression was conducted by including endotypes and other clinically relevant factors into the model. Of interest, patients with inflammopathic (OR 2.4, 95% CI 1.4–4.1, p = 0.001) and coagulopathic endotypes (OR 1.9, 95% CI 1.1–3.1, p = 0.014) had higher odds of having an adverse outcome compared to those with the adaptive endotype (**Supplementary Table 3**).

### Endotype Transitions

**Figure 2:**
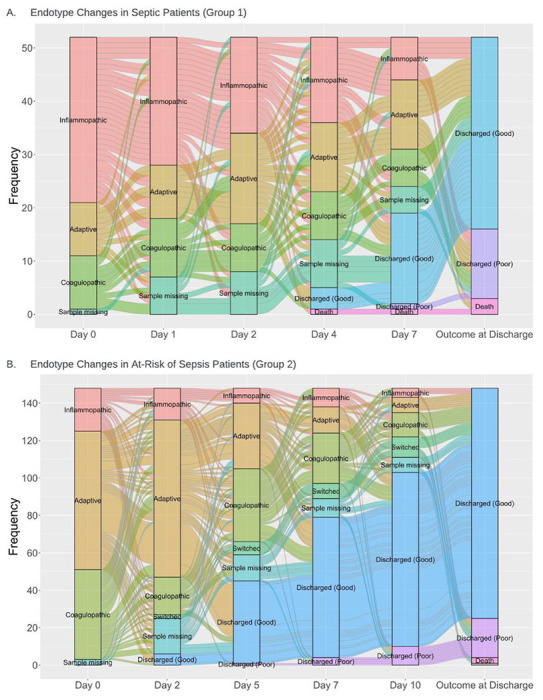
Alluvial Plots of Immunological Endotypes as they Change Over Time in Septic Patients (Group 1) and At-Risk of Sepsis Patients (Group 2). Measurements for both the septic (n=52, Group 1) and at-risk patients (n=145, Group 2) were obtained only from the second cohort (INF-06). 74% of patients changed endotypes during their hospitalization, 19% remained adaptative, 3.5% inflammopathic, and 3.5% coagulopathic. “Switched” is defined as those that transitioned into sepsis.

Figure 2 illustrates endotype distributions and transitions over time until death or hospital discharge. Measurements for both the septic (n = 52, Group 1) and non-septic patients (n = 145, Group 2) were obtained only from the second cohort (INF-06). 61 patients had at least one missing value, with 20% of data missing secondary to declined blood draw and 7% due to inadequate samples, labeling errors, or staff unavailability. Endotypes changed in 57.5% of patients during their hospitalization; of the remaining, 19% remained adaptative, 4% inflammopathic, and 3% coagulopathic.

We assessed pooled outcome data between septic and non-septic patients obtained after the last collected endotype measurement (Table 4). Based on similar clinical behavior and worse overall clinical outcomes, we also pooled inflammopathic and coagulopathic endotypes. In most cases, the final endotype assessment was drawn on day 7 or 10 of ICU admission. Patients who remained adaptive (n = 60) had overall better prognosis. Those who remained inflammopathic or coagulopathic had worse overall outcomes. There were non-significant differences between those who transitioned either from or to adaptive endotype.

### Endotypes and Predicted Severity

**Figure 3:**
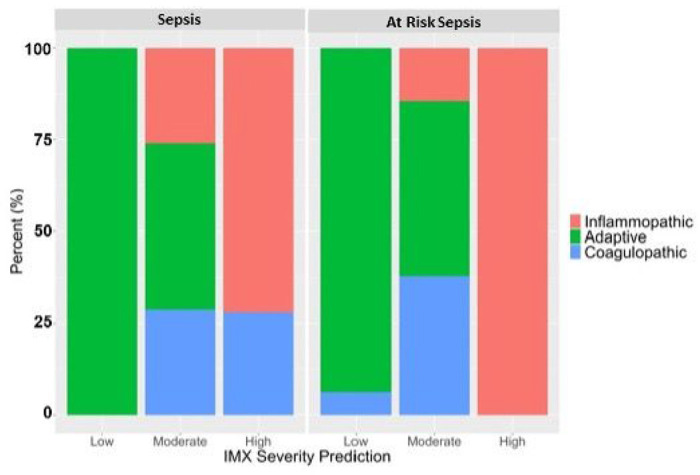
Immunological Endotypes of Sepsis and At-Risk Sepsis Patients Based on their Risk of Mortality using the IMX-SEV Severity Prediction Model. Sepsis (N=377) and At Risk of Sepsis (N=154) patients were stratified based on their mortality prediction model and assigned to either adaptive, inflammopathic, or coagulopathic. Importantly, patients stratified into the low risk of mortality using the IMX severity index were uniformly adaptive, while patients assigned to high risk of mortality were near uniformly inflammopathic or coagulopathic, regardless of whether they were septic or at risk of sepsis.

To better control for disease severity when comparing endotypes, we employed IMX-SEV-3 and found that endotypes were imbalanced across severity metrics (Fig. 3). Patients predicted to be low severity (n = 54), independent of their ICU admission cause, were near universally adaptive (98%): only one patient expressed a coagulopathic endotype while the patients with moderate severity prediction by IMX-SEV-3 continued to favor adaptive versus inflammopathic and coagulopathic endotypes (septic cohort: 45 vs 26 vs 29%, respectively; non-septic cohort: 48 vs 15 vs 38%, respectively). In contrast, those patients with high severity prediction based on IMX-SEV-3 were inflammopathic or coagulopathic in the septic (72% vs 28%) and inflammopathic in the non-septic (100% vs 0%) cohorts. We noted that inflammopathic (n = 69) and coagulopathic patients (n = 25) with a high risk of predicted mortality by IMX-SEV-3 appeared clinically similar, with nonsignificant differences in SOFA score, secondary infection, CCI, adverse outcomes, or mortality. The only noted difference was that inflammopathic patients demonstrated significantly higher plasma IL-6 concentrations than their coagulopathic counterparts (1,870 vs 642 pg/ml, p < 0.01; **Supplementary Table 3**).

## Discussion

### Key findings

This prospective study demonstrates that patients both presenting with sepsis and without sepsis can be characterized according to transcriptomic signatures of both endotype and severity from the point of ICU admission through to discharge.

### Context

Recent advances in sepsis endotyping research benefit from standard comparisons between studies, as advocated by De Merle et al [7]. By observing endotypes in other septic and non-septic patients, we join efforts to redefine sepsis nosology as a heterogenous condition that shares characteristics across the spectrum of critical illness [9]. Semantically, we choose the term endotype to highlight distinct pathobiological mechanisms amenable to targeted interventions to contrast against clinically observable phenotypes [7].

Our study shares commonalities and differences with others. The MARS consortium was first to investigate and validate patient endotypes in sepsis and identified four groups (MARS1-4) from the expression of 140 genes in the blood from 787 septic patients [22]. Genes were identified using unsupervised clustering techniques. The authors assessed mortality using values drawn at a single time point within 24 hours of admission and examined the biological plausibility of the identified genes known to cytokine signaling, cell proliferation, lymphocyte and metabolic pathways, among others. They did not examine changes in gene expression over the course of illness nor was there long-term follow up.

Endotypes detected within hours of patient presentation were also found to carry prognostic significance. Using available PCR technologies, Baghela et al validated five distinct gene expression profiles across several hospital systems, minimizing their findings to a group of 40 genes that clustered patients into *Neutrophilic-Suppressive, Inflammatory, Innate-Host-Defense, Interferon*, and *Adaptive* [18]. Despite overlapping terms with the current study, the classifiers used separate gene profiles, with only *ARG1* appearing in both models. The differences in models could be due to differences in cohorts or in the classification techniques used to derive the groups [12, 23]. Several other authors have also reported immune dysfunction using one-time blood draws [24, 34, 35].

Few studies, however, have analyzed gene expression profiles at different points during admission. In patients expected to require at least 72 hours of mechanical ventilation, a follow-up study of the PREVAIL trial, samples collected at days 1, 3, 6, 14, 21, and 28 were used to differentiate septic and non-septic patients using a novel scoring mechanism [21]. While they demonstrated changes in gene expression profiles through patient admission, they did not analyze outcomes. Similar global changes were noted in patients with community-acquired pneumonia and fecal peritonitis during admission [36].

### Current Work

Our study recapitulates observations about the 33-mRNA endotypes shown in previous investigations, but here using surgical patients and longitudinal data [20, 23, 37]. In contrast to these investigations, however, we found that inflammopathic and coagulopathic patients had more similarities than differences, perhaps representing a single endotype. When pooled together, we showed that patients who presented with inflammopathic or coagulopathic endotype had increased incidence of adverse outcomes and secondary infections, and trended towards increased mortality, regardless of their ability to transition to an adaptive endotype. Meanwhile, those that remained adaptative trended towards reduced 30- and 90-day mortality. These findings are similar to those described by Wong et al. who measured a binary endotype on day 1 and 3 of admission in pediatric patients [38]. In their study, Endotype A had worse outcomes regardless of transition to the more benign Endotype B and had less response to steroids. This suggests a possible steroid-responsive version of sepsis. Our study further corroborates evidence that septic patients may benefit from a personalized approach.

A second novelty of this study is that we were able to both identify and track changes in gene expression profile and severity scores over the course of a patient illness through to discharge. While admission endotype appeared to be the strongest predictor of outcomes, given the extensive crossover noted between days 2 and 7, we demonstrate that it may be valuable to continue assessing gene expression profiles, rather than focus on single timepoint. This permits monitoring for resolution of immunologic dyscrasia, severity of condition, as well as possible responses to therapy.

In addition to analyzing septic patients, we also included a non-septic, critically ill cohort. While inflammopathic patterns had higher rates of secondary infections regardless of sepsis status, there were no overall changes in mortality or poor discharge disposition. Interestingly, both inflammopathic and coagulopathic subjects in the high-severity risk category had similar outcomes. These results may contribute to the understanding of sepsis as a part of a spectrum of critical illness rather than a separate entity.

Finally, this study applied the endotyping signature in a surgical cohort, while prior evaluations have mostly been in medical, bacterial sepsis or COVID-19 patients [9, 18, 22, 35, 36, 38]. A recent report suggested the potential for endotypes to underpin different forms of critical illness [9]: a possibility that an *‘inflammopathic’* COVID-19 patient may be similar to an *‘inflammopathic’* surgical sepsis patient in molecular pathophysiology, further contributing to the idea of sepsis as a critical illness subtype.

### Limitations

We note several limitations to our study. First, this study was performed at a single institution with a predominately Caucasian patient population and may lack generalizability. However, both IMX-SEV-3 and the endotyping classifier have been validated multiple times in external hospitals with similar results [23, 33, 37]. Second, our non-septic cohort was broadly defined and with lower overall APACHE II scores. Age, gender, and Charlson comorbidity index were similar between the cohorts. However, when controlling for high-severity risk, we noted similar demographic and patient characteristics between the cohorts. Third, the majority (86%) of septic patients were derived from the initial cohort. These patients generally had higher SOFA scores and rates of CCI, with similar discharge disposition, complications, and mortality to the septic patients recruited in the second cohort. Fourth, as the first cohort was recruited from 2015 until 2020, there is the possibility of data drift, though standard of care for septic patients did not change during that period for our institution. Fifth, the multiple time series population contained only 196 patients, limiting our ability to draw conclusions based on trends and outcomes. Sixth, our findings regarding outcomes in final endotype measurements may not be representative of their endotype closer to the outcome measure, as day 10 measurements may have less impact on 30- and 90-day mortality. Finally, this paper did not seek to investigate the biological underpinnings of the mRNAs used in the two classifiers and their relation to pathophysiology; this has been done elsewhere [19, 23].

### Future Directions

Results from this study and others could assist in paving the way for personalization of sepsis treatment. By monitoring heterogenous, pathophysiologic responses to therapy, clinicians and researchers may be able to “divide and conquer” the sepsis syndrome and perhaps redefine sepsis along a spectrum of critical illness rather than as a separate entity. Current work into both immunosuppressant and immunostimulant therapies would benefit from targeting specific endotypes. The results of this study may be incorporated into randomized controlled trials or advanced causal analysis techniques employing observational data. From a prognostic standpoint, the conduct of similar endotyping on patients following discharge could also inform our clinical outreach efforts in diverting resources to those with greater follow up needs.

## Conclusion

Critically ill surgical patients with and without sepsis express different immunological endotypes. These endotypes are dynamic across a patient’s admission, are associated with distinct outcomes and transitions between them may inform patient prognosis and care. Endotyping may prove useful for selecting targeted therapeutics in the critically ill for personalized care management.

## Figures and Tables

**Table 1:** Patient Demographics at Enrollment.

	At Enrollment		
Variable	Septic (N = 377)^1^	At-Risk (N = 145)^1^	p-value^2^
**Age (yr)**	58.9 (15.4)	57.4 (19.4)	0.39
**Male**	202 (53.6%)	92 (63.5%)	0.04
**Race**			
African American	38 (10.1 %)	10 (7%)	0.02
Asian	2 (0.5%)	0 (0%)	
Other	2 (0.5%)	6 (4.2%)	
White	333 (88.8%)	127 (88.8%)	
Missing	2	2	
**WBC(x1000/mm^3^)**	17.9 (8.5)	12.9 (5.5)	< 0.001
Missing	1	6	
**Neutrophils (%)**	80.7 (12.9)	76.8 (15.3)	0.12
Missing	30	102	
**Lymphocytes (%)**	5.1 (4)	11.7 (8.1)	< 0.001
Missing	30	102	
**Lymphocytes (x1000/mm^3^)**	0.8 (0.5)	1.2 (0.8)	< 0.001
Missing	30	102	
**CRP (mg/L)**	194.4 (98.7)	161.4 (146.3)	0.55
Missing	194	137	
**IL-6 (pg/mL)** ^3^	738.6 (1,807)	148.7 (313.7)	< 0.001
Missing	3	2	
**SOFA Score**	6 (4)	3 (3.2)	< 0.001
Missing	3	0	
**Charlson Comorbidity Index**	3.2 (2.7)	2.7 (2.5)	0.03
Missing	2	1	

1 Mean (SD); n (%)

2 Welch Two Sample t-test; Pearson’s Chi-squared test; Fisher’s exact test

3 Values represent samples obtained within 24 hours post enrollment

**Table 2:** Clinical Outcomes, Endotypes, and Severity Predictions.

Variable	Septic (N = 377)^1^	At-Risk (N = 145)^1^	p-value^2^
**Secondary Infection**	114 (30.2%)	12 (8.3%)	< 0.001
**CCI**	122 (32.4%)	10 (6.9%)	< 0.001
**Adverse Outcome**	213 (56.7%)	40 (27.8%)	< 0.001
Missing	1	1	
**Poor Discharge Disposition**	152 (40.4%)	23 (16%)	< 0.001
Missing	1	1	
**In-Hospital Mortality**	28 (7.4%)	3 (2.1%)	0.02
**30-Day Mortality**	38 (10.2%)	6 (4.1%)	0.03
Missing	4	0	
**90-Day Mortality**	61 (16.8%)	8 (5.5%)	< 0.001
Missing	13	0	
**Endotype**			< 0.001
Adaptive	151 (40.1%)	74 (51%)	
Coagulopathic	97 (25.7%)	48 (33.1%)	
Inflammopathic	129 (34.2%)	23 (15.9%)	
**IMX-SEV Severity**			< 0.001
**Risk Band**			
Low	38 (10.1%)	16 (11 %)	
Moderate	250 (66.3%)	124 (85.5%)	
High	89 (23.6%)	5 (3.5%)	

1 n (%)

2 Pearson’s Chi-squared test

CCI: Chronic Critical Illness; Adverse Outcome is defined as cumulative incidence of in-hospital, 30- and 90-day mortality, development of CCI, and poor discharge disposition.

**Table 3:** Endotypes and Outcomes on ICU Admission.

	Septic (N = 377)				At-Risk (N = 145)			
Variable	Adaptive (N = 151)^1^	Coagulopathic (N = 97)^1^	Inflammopathic (N = 129)^1^	p-value^2^	Adaptive (N = 74)^1^	Coagulopathic (N = 48)^1^	Inflammopathic (N = 23)^1^	p-value^2^
**Secondary Infection**	30 (19.9%)	36 (37.1%)	48 (37.2%)	< 0.01	4 (5.4%)	2 (4.2%)	6 (26.1%)	< 0.01
**CCI**	40 (26.5%)	38 (39.2%)	44 (34.1%)	0.1	2 (2.7%)	2 (4.2%)	6 (26.1%)	< 0.01
**Adverse Outcome**	63 (41.7%)	63 (65.6%)	87 (67.4%)	< 0.001	18 (24.7%)	10 (20.8%)	12 (52.2%)	0.02
Missing	0	1	0		1	0	0	
**Poor Discharge Disposition**	49 (32.5%)	42 (43.8%)	61 (47.3%)	0.03	12 (16.4%)	7 (14.6%)	4 (17.4%)	0.95
Missing	0	1	0		1	0	0	
**In-Hospital Mortality**	7 (4.6%)	6 (6.2%)	15 (11.6%)	0.07	2 (2.7%)	1 (2.1%)	0 (0%)	> 0.99
**30-Day Mortality**	11 (7.4%)	9 (9.5%)	18 (14%)	0.19	4 (5.4%)	1 (2.1%)	1 (4.4%)	0.74
Missing	2	2	0					
**3-Month Mortality**	22 (15.2%)	16 (17%)	23 (18.4%)	0.78	5 (6.8%)	2 (4.2%)	1 (4.4%)	0.89
Missing	6	3	4					
**IMX-SEV Severity Risk Band**				< 0.001				< 0.001
Low	38 (25.2%)	0 (0%)	0 (0.00%)		15 (20.3%)	1 (2.1%)	0 (0%)	
Moderate	113 (74.8%)	72 (74.2%)	65 (50.4%)		59 (79.7%)	47 (97.9%)	18 (78.3%)	
High	0 (0.0%)	25 (25.8%)	64 (49.6%)		0 (0%)	0 (0%)	5 (21.7%)	

1 Mean (SD); n (%)

2 Kruskal-Wallis rank sum test; Pearson’s Chi-squared test; Fisher’s exact test

CCI: Chronic Critical Illness; Adverse Outcome is defined as cumulative incidence of in-hospital, 30- and 90-day mortality, development of CCI, and poor discharge disposition.

**Table 4:** Change in Endotypes over Time in ICU and Subsequent Outcomes.

	Endotype Change				
Variable	Adaptive to Adaptive (N = 60)^1^	Adaptive to I/C* (N = 24)^2^	I/C to Adaptive (N = 38)^3^	I/C to I/C (N = 74)^4^	p-value^5^
**In-Hospital Mortality**	0 (0%)	2 (8.3%)	1 (2.6%)	3 (4.1%)	0.15
**30-Day Mortality**	1 (1.7%)	4 (16.7%)	2 (5.3%)	7 (9.5%)	0.06
**90-Day Mortality**	2 (3.3%)	5 (20.8%)	3 (7.9%)	9 (12.2%)	0.07
**CCI**	1 (1.7%)	1 (4.2%)	5 (13.2%)	10 (13.5%)	0.04
**Poor Discharge Disposition**	7 (11.7%)	7 (30.4%)	8 (21.1%)	16 (21.9%)	0.2
Missing	0	1	0	1	
**Total ICU LOS (days)**	2 (1, 4)	6.5 (2, 8)	4 (2, 8)	5 (2, 11)	< 0.001
Missing	0	2	0	0	

* I/C (Inflammopathic/Coagulopathic), ICU (Intensive Care Unit), LOS (Length of Stay)

1 First and last endotypes are both Adaptive

2 First endotype was Adaptive; Last endotype was Inflammopathic or Coagulopathic

3 First endotype was Inflammopathic or Coagulopathic; Last endotype was Adaptive

4 First and last endotypes are both Inflammopathic or Coagulopathic

5 Fisher’s exact test; Kruskal-Wallis rank sum test

## Data Availability

The complete raw datasets generated and/or analysed during the current study are maintained and are available at the UF Clinical and Translational Science Institute Biorepository (https://www.ctsi.ufl.edu/research/laboratory-services/ctsi-biorepository-2/scirc-specimens-archive/). Requests for access to the data are made to the Biorepository directly who will provide a complete deidentified dataset containing both the clinical and transcriptomic data upon request (27).

## Abbreviations

ALC: Absolute Lymphocyte Count
APACHE II: Acute Physiology And Chronic Health Evaluation
CCI: Chronic Critical Illness
ICU: Intensive Care Unite
SD: Standard Deviation
SOFA: Sequential Organ Failure Assessment
WBC: White blood cell
IL-6: Interleukin-6
